# Parkinson’s disease and immune system: is the culprit LRRKing in the periphery?

**DOI:** 10.1186/1742-2094-9-94

**Published:** 2012-07-09

**Authors:** Elisa Greggio, Laura Civiero, Marco Bisaglia, Luigi Bubacco

**Affiliations:** 1 Department of Biology, University of Padova, Via Ugo Bassi 58/B, Padova, 35121, ITALY

**Keywords:** Parkinson’s disease, Leucine-rich repeat kinase 2 (LRRK2), Neuroinflammation, Cytokines

## Abstract

Leucine-rich repeat kinase 2 (LRRK2) is a large multidomain kinase/GTPase that has been recently linked to three pathological conditions: Parkinson’s disease; Crohn’s disease; and leprosy. Although LRRK2 physiological function is poorly understood, a potential role in inflammatory response is suggested by its high expression in immune cells and tissues, its up-regulation by interferon γ, and its function as negative regulator of the immune response transcription factor NFAT1. In this review we discuss the most recent findings regarding how LRRK2 could be a player in the inflammatory response and we propose a scenario where the detrimental effects mediated by Parkinson’s disease LRRK2 mutations may initiate in the periphery and extend to the central nervous system as a consequence of increased levels of pro-inflammatory factors permeable to the blood brain barrier.

## Introduction

In the last 15 years, the contribution of genetics to our understanding of complex disorders has been tremendous. One example is Parkinson’s disease (PD), a neurodegenerative disorder thought to be exclusively triggered by environmental factors until 1997 when mutations in the *SNCA* gene, coding for alpha-synuclein, were identified in families with Mendelian inheritance of the disease [1]. Subsequent to that discovery, not only have other loci been linked to Mendelian forms of PD, but the more recent genome-wide association studies (GWAS) have highlighted how important genetic contribution can be for the development of sporadic disease (reviewed in [2]). One of the key genes in PD is *LRRK2*, mutations in which have been identified in a large number of families affected by an inherited form of parkinsonism with clinical presentation and disease onset very similar to the more common sporadic syndrome [3,4]. More recently, common genetic variation at the *LRRK2* locus have been shown to modulate the risk for sporadic PD [5,6] strengthening the links between this gene and the neurodegenerative process underlying PD. Interestingly, two recent GWAS found that common LRRK2 variants are also associated with Crohn’s disease (CD) [7], an inflammatory bowel disease, and leprosy [8], a chronic infectious disease caused by *Mycobacterium leprae*.

PD is characterized by the degeneration and death of dopaminergic neurons in the *substantia nigra pars compacta* (SNpc) that project into the striatum, where specialized neuronal circuits control body movement. Another pathological hallmark of the disease is the presence of intracellular, alpha-synuclein-containing aggregates termed Lewy bodies in surviving neurons [9]. Microglial activation is commonly observed in postmortem PD brain tissue, suggesting that inflammation may play a role in disease [10], although it is unclear whether this inflammation is causative or a secondary effect of upstream, earlier pathological events. Since its identification in 2004 as a gene linked to familial PD, the most obvious tissue to study LRRK2 function in was the brain, and in particular within the dopaminergic neurons that degenerate in PD. Therefore cellular and animal models have been conceived and studied according to these precepts. However, the low expression of LRRK2 in the SNpc compared to other brain areas and peripheral tissues or organs [11-14] has hindered the investigation of its normal and pathological role and, at present, there is no clear function attributable to LRRK2 in relation to dopaminergic neurons of the SNpc.

What do PD, CD, and leprosy have in common? Inflammation appears a common theme in all three diseases and the recent literature pinpointing a role of LRRK2 in immune response pathways [15-20] hints that LRRK2 dysfunction in PD may involve the immune system. Recent findings [16,17] showed that LRRK2 is highly expressed in human peripheral blood mononuclear cells and macrophages, is up-regulated by interferon γ (INF-γ), and its expression is increased by exposure to microbial structures or viral particles, strongly suggesting a role in immune response pathways. In the brain, LRRK2 activity is increased post-transcriptionally in the microglia of LPS-treated mice [20]. Furthermore, an elegant study by Liu and collaborators [19] demonstrated that LRRK2 is a negative regulator of the Nuclear Factor of activated T-cells (NFAT) and observed that LRRK2^−/−^ mice display abnormal sensitivity to experimentally induced colitis [19]. Here, we review the current knowledge of LRRK2 function and how this may relate to a role in inflammatory response. We will also advance the hypothesis that LRRK2 dysfunction in the periphery may affect the central nervous system and trigger the neurodegenerative process observed in PD.

### LRRK2 and Parkinson’s disease

Interest in studying LRRK2 biology started in 2004 when two groups independently reported that point mutations in the *LRRK2* gene are linked to dominantly inherited forms of PD closely resembling the idiopathic syndrome [3,4]. LRRK2 is a large, 286 kDa protein containing an enzymatic core comprising a ROC (Ras Of Complex proteins)/GTPase, a COR (C-terminus of ROC) and a serine threonine kinase domains, as well as a number of predicted protein-to-protein interaction domains at both terminals [21]. LRRK2 is an active kinase *in vitro*, with robust autophosphorylation and phosphorylation of model peptides [22-24], while measurements of its GTPase activity has been more challenging due to the scarce protein yields obtained after purification [25-27]. PD mutations cluster within the two enzymatic domains [28], suggesting that altered signaling may be implicated in the disease. We have previously shown that kinase activity is required for mutant proteins to be neurotoxic and to aggregate, at least in neuronal cell models [23], hinting that alteration of LRRK2 signaling might have pathological implications. However, only one mutation, the G2019S, clearly increases kinase activity *in vitro*, while other mutations do not display a significant effect [28]. Since blocking kinase activity prevents the toxicity of mutant LRRK2 observed in primary neurons [29], inhibition of LRRK2 kinase function holds great therapeutic expectations. It is not clear, however, why toxicity is also prevented by blocking kinase activity in mutants that are not linked to increased kinase activity [23,29]. One possibility is that the kinase domain acts as a *cis* regulator upstream of the GTPase/ROC domain and ROC is the signal output of LRRK2. In support of this hypothesis, it has been shown that the kinase phosphorylates ROC at multiple sites [30-32], suggesting that the GTPase activity is finely tuned by the kinase activity. Despite the significant commitment in the field, no robust physiological substrate of LRRK2 kinase activity has been reported to date, posing the question whether the LRRK2 kinase domain, other than mediating autophosphorylation, is relevant *in vivo*. Of interest, the potent LRRK2 inhibitor IN-1 [33] completely abolishes LRRK2 phosphorylation at Serine 910 and 935, causing loss of 14-3-3 s binding and accumulation of LRRK2 into large intracellular filamentous or punctate structures [34,35]. Interestingly, LRRK2 proteins with pathological mutations in the ROC-COR domain, but not with the G2019S mutation in the kinase domain, are unable to bind 14-3-3 s, display impaired phosphorylation of S910/S935 and are more prone to oligomerize than wild-type proteins when overexpressed in cell lines [23,34-36]. Mutations in LRRK2 are inherited in a dominant manner but whether the pathological mechanism is driven by a gain of function (that is, altered enzymatic activity) or by a loss of function through dominant negative or haplotype insufficiency effect, is still unclear. One possibility is that proteins with pathological mutations, which have a higher tendency to aggregate *in vitro*, sequester functional wild-type proteins resulting in loss of LRRK2 physiological function. This may also explain while LRRK2 mice models of mutant LRRK2 are healthy and do not display cellular, functional, or motor abnormalities rather than subtle defects in dopaminergic neurotransmission [37-39]. Interestingly, knock-out (KO) models display renal abnormalities at old age associated with impaired autophagy, alpha-synuclein aggregation, and activation of the inflammatory response in kidneys [40-42]. Although this phenotype is only observed in the kidneys and not in the brain, the relevant site of the neurodegenerative process, it is possible that the lower expression levels of LRRK2 in the nervous system compared to the renal tissue causes only subtle pathogenic effects, which may reflects, in turn, the late onset of the disease. If mutant LRRK2 associated PD occurs via loss of function, then LRRK2^−/−^ mouse would be an accelerated model of the LRRK2-linked disease with respect to the mutant transgenic models.

LRRK2 has been suggested to play a role in the control and maintenance of neurite length [43-46], in vesicle trafficking at the presynaptic site [47], in activation of apoptosis through interaction with death adaptor Fas-associated protein with death domain (FADD) [48], and in regulation of autophagy pathways [40,49-51]. However, the detailed interactome and precise signaling cascade(s) that are orchestrated by LRRK2 are still missing pieces in the function/dysfunction jigsaw puzzle for this protein.

### LRRK2 as an immune-response regulator

Recent experimental findings point to a clear role for LRRK2 in the immune system. Analysis of LRRK2 mRNA expression in different tissues indicates that the highest levels of expression occur in immune cells, particularly in B cells, macrophages, and dendritic cells, with lower levels in T cells [16,17,52]. LRRK2 is recruited near pathogens during bacterial infection [17], is up-regulated upon exposure to microbial and viral particles [16,53] and LRRK2 deficiency impairs reactive oxygen species production during phagocytosis [17]. Interestingly, LRRK2 expression is significantly induced upon INF-γ stimulation in peripheral blood mononuclear cells (PBMCs) [17], in primary microglia from an R1441G transgenic LRRK2 mouse [53], and elevated INF-γ levels are pathological hallmark of CD [54]. INF-γ is a cytokine that coordinates a variety of cellular programs through transcriptional regulation of immunologically relevant genes [55]. One possibility is that high levels of INF-γ, as observed in CD, result in up-regulation of LRRK2 function. LRRK2 has been suggested to activate NF-κB signaling in HEK293T cells [17], although others did not observe a similar effect [19]. NF-κB signaling plays a pivotal role in regulating the immune response to infections [56] and chronically elevated NF-κB signaling in PBMCs may contribute to CD onset. These results suggest that LRRK2 is an INF-γ inducible gene, linking to the observation that patients with CD display a six-fold increase of LRRK2 levels [17]. Overall, these findings indicate that induction of INF-γ expression by CD4+ cells consequent to microbial infection causes activation of a set of immune response genes including LRRK2, the expression of which activates NF-κB signaling to coordinate the immune response process.

Another interesting piece of evidence linking LRRK2 to immune system comes from a recent report by Liu and co-workers [19]. Starting from an *in silico* analysis of high throughput RNAi databases, they found that the *Drosophila* LRRK2 ortholog was in a list of genes controlling NFAT1 nuclear translocation. They demonstrated elegantly that LRRK2 is a negative regulator of NFAT1 transcriptional activity by inhibiting its nuclear translocation via association with the NRON complex, which physically inhibits NFAT1 nuclear translocation [19]. Transcriptional activity of NFAT1 orchestrates the expression of a number of proinflammatory cytokines such as IFN-γ, interleukine-1 (IL-1), and tumor necrosis factor alpha (TNFα). Elevated levels of TNFα are commonly observed in CD and anti-TNFα therapy has been shown to be effective at ameliorating disease symptoms [57]. Therefore, LRRK2 may function as a transcription regulator of immune related pathways by modulating the function of transcription factors such as NFAT1 and NF-κB. Interestingly, NF-κB activation by LRRK2 is independent of its kinase activity as the kinase inactive K1906M mutant and the pathological hyperactive G2019S mutant display comparable ability to activate NF-κB as the wild-type protein [17]. A similar observation was made for NFAT1, whose nuclear translocation is inhibited by LRRK2 in a kinase-independent manner [19]. The single nucleotide polymorphism associated with CD (rs3761863) [7] leads to a T2379M amino acid substitution, located in the central part of the WD40 domain at the C-terminal end of LRRK2. Protein containing the M2379 variant exhibits a decrease in protein stability compared to the T2379 variant [19], supporting the notion that the amount of LRRK2 rather than its catalytic activity is important for its immune related function. Furthermore, T2379M carriers have slightly increased NFAT1 activation [19] with consequent elevation of cytokine production, which may explain the increased risk associated with CD. Overall these observations point to a model where LRRK2 acts as scaffold to orchestrate the activation of immune response signaling cascades culminating in gene transcription and that the levels of LRRK2, rather than its catalytic activity, are important for complex formation.

### Neuroinflammation as a key player in PD

Microglia are macrophages resident in the brain and represent the first line of defense of innate immune system. While most of microglia functions are protective, there is mounting evidence that chronically activated microglia and astrocytes contribute to PD (reviewed in [58]) and that peripheral inflammation increases microglia activation with consequent damage of the nigrostriatal dopaminergic circuit (for a review see [59]).

Proinflammatory cytokines, such as IFN-γ, TNFα, and IL-1 coordinate the action of microglia and PD patients have been found to possess elevated levels of TNFα and IFN-γ in cerebrospinal fluid and postmortem brain tissue [60,61].

A recent report demonstrated that a single systemic administration of lipopolysaccharide (LPS) results in microglial activation and neuroinflammation that persists long after peripheral events have abated, and that this induces a delayed and progressive loss of DA neurons in the SNpc [62]. In agreement with another study indicating that very little peripheral LPS enters to the brain due to the poor passage through the BBB [62,63], the authors demonstrated that TNFα produced in the periphery after systemic LPS administration is transported into the brain through a well-documented TNFα-receptor-dependent mechanism [64]. Interestingly, parkin-deficient mice, a genetic model of recessive parkinsonism [65], display DA neuron degeneration when systemically exposed to LPS treatment, indicating that sustained neuroinflammation cooperates with genetic predisposition to trigger neuronal degeneration [66]. Another elegant paper by Moehle and collaborators shows that mice injected intracranially with LPS display robust LRRK2 induction at the post-transcriptional level [20]. Furthermore, they observed that pharmacological or genetic LRRK2 inhibition reduces TNFα release by the microglia, indicating that in this population of cells the kinase activity might be important in mediating the inflammatory process. In agreement with these findings, a recent paper by Gillardon and co-authors reports increased LRRK2 expression and TNFα secretion upon LPS stimulation of primary microglia from R1441G LRRK2 transgenic mice [53].

How increased peripheral pro-inflammatory cytokines enter the brain, or whether they are released *in situ* by activated microglia, and how this links to degeneration of DA neurons in the SNpc remains unclear. One possibility is that DA neurons are more susceptible to a particular subset of cytokines, as for example TNFα, IFN-γ, or IL-1β, which have been shown to selectively damage DA neurons [67]. Accordingly, TNFα receptor 1 is expressed in dopaminergic neurons [68] and its expression level is increased in PD [69]. Another possibility is related to the pronounced sensitivity of DA neurons to oxidative insults, produced by activated microglia, due to the presence of DA itself and because the SNpc has lower levels of glutathione (GSH) compared to other brain regions [69]. In this situation, pro-inflammatory cytokines represent the trigger event that activates microglia. Remarkably, SNpc contains 4.5 times as much microglia when compared to other brain regions [70], making this region particularly susceptible to inflammatory insult. At the same time, the density of astrocytes is low in the SN compared to brain areas which are less affected in PD [69], leading to the possibility that an environment with reduced numbers of astroglia might contribute to the regional specificity of the disorder.

### Could LRRK2 function in immune response relate to PD etiology?

A recent study by Mutez and collaborators shows alterations in the transcriptional profile of blood mononuclear cells from PD patients with LRRK2 mutations. In particular, they observed dysregulated interleukin signaling and TGF-β signaling, further supporting the notion that PD also involves neuroinflammatory processes and peripheral immune infiltration [71]. Moreover, these observations imply that analyzing selected pathways that become altered in peripheral cells may help to identify and delineate the early stages of PD, using dysregulated genes as molecular markers.

The key question is whether PD-linked LRRK2 mutations impair LRRK2 function in DA neurons or, instead, in immune cells with a deleterious impact on DA neurons as a secondary effect. As previously emphasized, proinflammatory cytokines, such as IFN-γ, may influence LRRK2 expression which, in turn, can activate the nuclear transcription factor NF-κB. Interestingly, not only was NF-κB activation detected within the SN of PD patients, but a marked co-localization of NF-κB and p65 with GFAP-positive activated astroglia and CD11b-positive activated microglia was also observed [69]. As discussed, it is still not clear whether PD LRRK2 mutations operate through a gain or loss of function. The latter mechanism implies that mutant proteins act in a dominant negative fashion, recruiting and neutralizing functional wild-type proteins. A possible mechanism for this is suggested by the dimeric nature of LRRK2 [72-75], and the observation that mutant LRRK2 displays a higher tendency to oligomerize [16,23,36]. In this scenario, a LRRK2 heterodimer containing mutant protein and displaying decreased physiological activity may impair LRRK2-dependent inhibition of NFAT1 nuclear translocation with a consequent increase of NFAT1 transcriptional activity and cytokines production. Abnormally elevated levels of cytokines may activate microglia and establish feed-forward loops that result in sustained inflammation. The CNS has traditionally been considered to have a privileged immune status, and is protected from the action of much of the immune system by the BBB. However, recent findings indicate that both innate and adaptive immune systems play critical roles in the pathogenesis of PD [69,76]. For example, it has been reported recently that there are higher densities of CD8+ and CD4+ T cells near DA neurons, in the brain of patients with PD than in healthy individuals [77]. Moreover, a subset of cytokines such as IL-1 has been shown to cross the BBB [78] and chronic systemic administration of IL-1 exacerbates DA neuron degeneration [78]. Therefore, subtle increases in the concentration of cytokines within the brain may have a long-term, cumulative effect on neuronal toxicity, which reflects the late onset of mutant LRRK2-linked PD. It has been shown that the BBB becomes damaged during age and leukocyte extravasation is observed in autoptic SNpc from PD patients [79,80]. A key issue that remains to be determined is whether LRRK2 function/dysfunction in PD involves enzymatic activity or not. Therapeutic strategies to prevent LRRK2-linked PD are directed at the most obvious target: kinase activity. However, if mutant LRRK2 acts by sequestering functional protein and amount of LRRK2 protein rather than its enzymatic activity is important for its physiological function, then inhibition of kinase activity may not be the most appropriate strategy. However, given that the common G2019S risk allele augments kinase activity, at least *in vitro*, and that kinase inhibition *in vitro* is protective against DA neuronal loss and attenuates the inflammatory response in the microglia, the true pathological situation is likely to be more complicated. In regard to this, it will be important to test whether transgenic mutant LRRK2 mice are more susceptible to experimentally induced colitis, similar to the LRRK2^−/−^ mouse, which would support a loss of function mechanism for PD mutations.

## Conclusions

In conclusion, recent findings have highlighted a clear role for LRRK2 in the immune system. Moreover, variations in the *LRRK2* gene are associated with increased risk of CD, an inflammatory disorder, reinforcing the notion that LRRK2 acts as a regulator of the immune system. The future key work in this regard will be to address whether LRRK2 dysfunction in PD is also linked to alterations in its immune function, and whether LRRK2 toxicity associated with PD occurs through a gain or loss of function in order to design the most appropriate therapeutic approach.

## Competing interests

The authors declare they have no competing interests.

## Authors’ contributions

EG, LC, MB, and LB conceived and wrote the manuscript. All authors read and approved the final version of the manuscript.
